# Social Context of Disabled Parenting

**DOI:** 10.1007/s11195-014-9349-5

**Published:** 2014-04-12

**Authors:** Monika Parchomiuk

**Affiliations:** Faculty of Pedagogy and Psychology, Institute of Pedagogy, University of Maria Curie Sklodowska, Narutowicza Street 12, 20-004 Lublin, Poland

**Keywords:** Parents with disability, Socialization, Social attitudes, Experience of motherhood and fatherhood, Poland

## Abstract

The article discusses parenting of individuals with disability. It was based on the perspective of barriers, which (next to the perspective of risk and the functional perspective) marks the nature of discourse this theme takes in the literature on the subject and in practice. The perspective of barriers, related to the social model of disability, emphasizes the social factors important for the quality of parenting of individuals with disability. These factors were analyzed on the basis of available research results with mothers and fathers with disability, and able-bodied individuals who are part of their professional and non-professional environment. The article draws attention to the specificity of socialization of individuals with disability and its significance for preparations for family roles, social attitudes towards disabled individuals’ parenting, and their determinants. It also presents disabled parents’ experiences and the importance of realizing their role of a mother or father.

## Introduction

Parenting is a social construct [1]. It means that social agreements are used to determine how parenting should be realized, to form values and norms which become the basis for its evaluation, and to prepare individuals to become parents, expecting at the same time that they will fulfill their parental responsibilities. There is a model of ideal parenting in social consciousness centered around the expectation that mothers and fathers should fulfill their parental tasks successfully. The concept of motherhood is built on expectations of complete devotion (mostly to the child), and taking care of the family members in need. Publications from the 80 s read as follows:

Mothers in modern Western societies are idealized as ‘natural’ caregivers for children and are held fully responsible for the good and bad that befalls their children. They are also liable for the moral reproduction of society through the nurturing of physically, emotionally and morally healthy children [2, p. 471].

It seems that this idea of motherhood still persists, alongside the fact that women are more and more often expected to reconcile successfully their motherly duties with professional self-realization. Similarly to these changes concerning women, social expectations of fathers also evolve. The dominant model of a breadwinner unengaged in protective issues—tackled by women—has changed. Fathers are more often expected to get involved in the care giving and upbringing sphere [3, 4]. The quality and scope of parents’ efforts to meet the social norms of ‘the ideal parenting’, and the impact of these actions on the parents themselves and on their children, depend on many factors. According to the ecological approach, there are three spheres of complex determinants: (1) children’s developmental needs (2) parent’s capability and personal resources to fulfill these needs (3) a broader social and economic context where parents function [Olsen, Clarke, in 5]. This perspective on parenting is now being postulated as useful in evaluating how disabled parents function.

Literature on the subject offers a rather disjointed discourse on disabled parents. It revolves around three perspectives, none of which seems to have been given ample scholarly attention: (1) the risk perspective (2) the functional perspective and (3) the barrier perspective. The first is the subject of research and theoretical analysis devoted to children of parents with disability; it is aimed at finding both biological and psycho-social hazards related to these parents’ deficits. The functional perspective encompasses studies on the impact of specific impairments. Most of these concentrate on difficulties (mainly practical) encountered by parents. Both perspectives follow the so-called medical model of disability. The social model is where the analysis from the barrier perspective are realized. These concentrate on environmental factors which determine the quality of disabled parenting, such as social attitudes towards procreation and parenting of individuals with disability, as well as barriers and difficulties resulting from no or inadequate support for these individuals in realizing their family roles. In the context of such analysis, grounded in the social model of disability, egalitarianism of disabled parents’ needs and equality of rights and privileges are postulated [5]. The social model of disability draws attention to the issue of parental competences which are assessed by the society. The significance of these competences should not be limited to the quality of realizing practical tasks (related to everyday activities, such as running a household, or giving care to a child); it should rather lie in the ability to function autonomically and responsibly [6]. Even when they need support in practical tasks, or ongoing support, disabled parents are still competent. They should retain some degree of independence in decision making, to be able to make important choices, predict the outcome and be responsible for the consequences, both from their own perspective, and from the perspective of dependent family members (especially children). This independence is an important personal skill, closely related to other personal qualities, such as autonomy, responsibility, sense of control, self-awareness and others. The abovementioned qualities are shaped across human lifespan, but they are heavily influenced by experiences from the time of socialization.

The following analysis aims to present complex social factors which form the context for realization of parental roles by individuals with disability. My own conclusions, grounded in studies of literature on the subject, but also those referring to my own research into social attitudes towards procreation, parenthood, maturity of individuals with disability constitute the basis for these analysis. They are grounded in and corroborated by references to other authors’ works published in journals on the subject. Analyzing social context of disabled parenting, I do not categorize on the basis of physical and intellectual limitations. Following the social model of disability, I assume that environmental factors play a significant role, alongside the individual (person’s with disorders) and social (persons’ from his/her environment) perception of how significant the limitations are (since this perception leads to certain attitudes and expectations). I am taking into account persons with lifelong-developmental and early acquired—disability. My analysis will concern (1) experiences of socialization important in shaping adult life skills and realizing parental roles (2) social attitudes towards procreation and parenting of individuals with disability (3) positive and negative experiences of mothers and fathers with disability resulting from individual and social factors. I see my analysis a voice in a discussion on parental competences of individuals with disability. Understanding complex social conditions for these competences is an introduction to realizing support for parents with disability, but also of able-bodied parents, caregivers, and specialists preparing children with disability to adult life.

## ‘Disabled’ Socialization: Experiences of Socialization Important in Shaping Adult Life Skills and Realizing Parental Roles

Disability, as much as parenting, may be perceived as a social construct. It means that society agrees on a specific way of understanding it, and postulates a way of evaluating it from the perspective of possibilities and limitations. The importance of possibilities and limitations (which result from the physical damage and which shape the identity of an individual with disability) is determined in the process of socialization, especially in the family. Socialization is understood here as the intentional and unintentional influences which affect an individual who is given tasks to perform and needs to obtain information on how to perform them; but it also denotes various ways in which the environment tries to fulfill this individual’s needs and teach them desirable competences.

Experiences of individuals with disability gained in their family of orientation are largely influenced by their parents’ psycho-social functioning, and with it, by their parents’ adaptation abilities and resources which are necessary to deal with the demands of raising a child with disability at various stages of living together. In this respect, parents may show deficits related to concentration and adoption of wrong attitudes: rejecting the child (keeping distance), patronizing, or being helpless. Complex consequences of such attitudes, especially if they are strong, include: not fulfilling the child’s multiple needs, which results in building their negative self-image dominated by the damage (defect), the sense of weakness and dependence on others; not developing autonomy, the ability of self-determination, optimism, self-efficacy, or other mental properties important for setting oneself life goals and achieving them, and for overcoming obstacles on the way; plus want of social competence [7, 8].

Socialization of some individuals with disability takes place in institutional conditions. This mainly concerns individuals with intellectual disability or mental illness. Conditions within these institutions limit the acquisition of life skills. Social isolation, routine and schematism of everyday life, combined with the inability of self-determination, are the main factors which hinder preparation for life outside, predominantly since they reduce the possibilities to develop the abovementioned competences. Individuals living in caring institutions do not have the family role models which represent certain values and norms regulating family life and show standards of behavior [6, 9]. An individual living in a specific institutional environment is deprived of the intergeneration message about family life. Similarly, these individuals do not experience support which is derived from intergeneration relationships offered in situations when a person undertakes a new social role (e.g. grandparents taking care of their grandchildren).

On the other hand, socialization in the family may be a source of negative family role models. It happens in families with problems in the emotional sphere, pathologies (e.g. addictions or violence), or where the needs of dependent family members (children, persons with disability, the elderly) are neglected. Such cases are more often found in families of persons with mild intellectual disability [10]. Studies with parents with intellectual disability who were deprived of their parental rights on the grounds of difficulties in fulfilling their role inform about their negative life experience: deprivation and being victims of violence in their childhood [11]. Cases of defective family models may also be found in able-bodied families. It seems, though, that these individuals are more likely to ‘work through’ the negative experiences related to how they functioned in the family, and activate compensation processes. It is easier for them to acquire competences by means of their own cognitive efforts: by observing the environment, gathering and studying the data, reaching rational conclusions, and by studying literature on the subject. They also have access to much wider support than individuals with disability [8].

Test results obtained by L. Marszałek [8] confirm the existence of a phenomenon which, referring to the title of the article, may be called ‘disabled socialization’. She used structured interview in her studies with three groups of respondents (1) women with physical disability aged 18–25, who are not yet married and do not have children (2) able-bodied women aged 18–25, who are not yet married and do not have children (3) and married women with physical disability aged 26-35. She claimed that women in groups 1 and 2 have significantly different experience of socialization. According to her, social environment (especially family members) form expectations which have negative impact on how women with disability function in marital and parental roles—they make it impossible or hard for women to take up these roles. I have been led to believe that a person with disability develops a “disabled identity” which impairs constructing individual and social identity; instead, a self-image dominated by limitations in various roles and life tasks evolves.

However, since it rarely happens that individuals with disability experience extreme deprivation of the social stimuli, they still receive information about the social role of a woman/man along with the values ascribed to it by the society. The image of motherhood, as an ‘ideal’ time in the life of a woman and a standard of adult life facilitating realization of social expectations related to sex, is present in the responses of women with disability [1]. The role of a ‘disabled individual’ accepted in the process of socialization leads to a conflict with these ideals and standards:

A disabled woman is aware of the fact that she does not fall within social stereotypes and ideas about a ‘real woman’, and that her physical features are perceived by the environment as limiting or making her incapable of fulfilling her role. […] the individual limits her aims and aspirations related to undertaking and realizing her role [8, p. 106].

On the basis of her study with women with disability who have not taken up their family roles, L. Marszałek [8] concludes that these women are significantly less willing to get married, and even less willing to become mothers than able-bodied women who have not taken up their family roles (98 % of able-bodied women and 61 % of women with disability claimed they wanted to become wives; 95 % of able-bodied women and 41 % of women with disability claimed they wanted to be mothers).

Among the reasons for this situation in the group of women with disability were their convictions that they were unattractive (a reason for not getting married) and unable to provide children with proper care. The same reasons were not mentioned in the control group, where mental traits were brought up by just a few respondents. More women with disability than able-bodied women believed that they were not prepared to take up family roles (56 vs. 11 %). They also perceived themselves as less competent in family roles: 39 % of women with disability and 2 % of able-bodied women felt they were incompetent to fulfill their role of a wife; 61 % of women with disability and 5 % of able-bodied women felt they were incompetent to fulfill their role of a mother. Analysis with variables concerning the nature of family experience have proven that overprotectiveness of family members and even peers was a factor correlated with a weak sense of competence to fulfill family roles [8]. Similar results were obtained by O. Prilleltensky [6], who conducted in-depth interviews with women with muscle dystrophy. The author found that few of those women believed they could lead a life similar to that of their able-bodied peers, and the ones who were pregnant had fears and doubts whether they would be able to fulfill their child’s needs. Their anxiety was undoubtedly also caused by limited access to information.

### Social Attitudes Towards Procreation and Parenting of Individuals with Disability

It seems that it is easier to answer the question what social attitudes towards disabled parenting are than to say which factors determine these attitudes. Undoubtedly, both answers will be only broad generalizations, since attitudes towards parenting, similarly to general attitudes towards individuals with disability, will differ among respondents and will be influenced by many factors.

In the course of research with various groups (students of pedagogy, students of medicine, specialists, and parents of intellectually disabled persons) it was found that the concept of parenting, analyzed as one of the aspects of broadly understood sexuality of individuals with disability, is evaluated negatively. Semantic differentials with concepts describing human sexuality in its physical dimension (e.g. body, sexual drive) and in its psycho-social dimension (e.g. marriage, parenting) were used. This tendency is most clearly visible in the case of parents with intellectual disability [12–15]. Similar tendencies are noticed by other authors who review and analyze literature on the subject [16–18]. The image of disabled parenting held by the society is dominated by negative aspects such as difficulties, lack of abilities and not fulfilling one’s role properly. During group interviews in a study by M. Starke [19], specialists who work with disabled parents (19 persons including social workers, special needs pedagogues, and psychologists) pointed to these individuals’ inability to understand the essence of parenting or learn the necessary skills, especially the emotional ones. The predominant limiting factor and the basis of all the difficulties is the disability itself.

Social attitudes of persons who are important in women’s life (their close ones, e.g. parents) and of the specialists who perform duties related to taking care of the mother and child and then of the family contain a wide spectrum of reactions and behaviors:

1. Devaluation of parenting when parents have a disability, which is visible in: suggesting abortion, or giving the child up for adoption, attempts at depriving parents of child support, looking for pathology and parental incapacity on the basis of how the child functions; patronizing, taking the attitude of disabilism (treating the person with disability as dependent, lacking intellect, and requiring help); discrimination visible in the fact that individuals with disability need to explain the reasons why they want to start a family;
2. Reactions of surprise in situations when one cannot reconcile their convictions about the nature of disability and the observed parenting skills of a person with disability^1^;
3. Acceptance, positive evaluations, efforts directed at providing rational support appropriate to the parents’ needs [6, 20–24].

Looking for factors related to the described attitudes (expressed in the negative aspects), one should also refer to the social perception of disability. “Disability is so closely associated with dependence and social isolation that it is hard for people to imagine a disabled individual at the centre of family life in the role of primary carer” [Wates, in 5, n. pag.]. “Women with disabilities are seen as dependent and in need of being taken care of, it is difficult for many to imagine how a mother with a disability can fill the caring and nurturing mothering role” [25], n. pag.] In my view, this image of disability plays a significant role in the way individuals are perceived in various social roles. Individuals with disability have problems with access to employment, and their life situation forces them to depend on various forms of public aid. Their parenting is seen as an additional burden on the society.

In a discussion on procreation of individuals with disability one should also refer to attitudes towards their sexuality. The latter is often perceived as flawed (especially in the context of inheritance), or non-existent. If one denies the presence of the sexual sphere of individuals with disability, or perceives it as a threat because of genetic determinism, it is difficult to talk about acceptance of procreation. Eugenics, which in some countries (the US, Scandinavia, Western Europe) has strong legal foundations and social consent—although officially in decline—has its impact on the way specialists and laypeople think about access to procreation. Hence the suggested solutions, like abortion, which are not always grounded in health considerations of women with disability.

Perhaps one of the factors underlying negative attitudes towards disabled parenting is inadequate knowledge about these parents’ experience, special and everyday needs, and about the significance of support [26].

In his discussion of the social image of disabled parenting, M. R. Reinikainen [27] speaks of a dualism: these individuals’ access to procreation is limited (by specialists in various fields), while parenting is treated as a sign of ‘normality’ (normal femininity and masculinity). On the basis of 62 diaries of women with disability who took part in the contest, the author concludes that parenting does not necessarily remove the stigma of abnormality caused by disability. If an individual with disability wants to start a family, they are subject to close scrutiny by the specialists who will often quote medical discussions on threats related to procreation, such as hereditary disorders, or fetal damage caused by parents’ medicalization. Although this type of discourse also concerns many able-bodied women who have contact with health institutions during procreation, women whose bodies are ‘abnormal’ are especially prone to experience stronger monitoring related to it [24].^2^ The intensity and impact of this discourse is clearly visible in mothers’ sense of guilt after giving birth to a genetically defective child, and their difficulty in accepting this child as a valuable human being [24]. This guilt is founded in the conviction that they did something for themselves, that they realized their desires and dreams, which means they were egoistic.

## Individuals with Disability in Family Roles: Positive and Negative Experiences of Mothers and Fathers with Disability Resulting from Individual and Social Factors

To present a broad perspective on the discussed theme it is necessary to show the importance of parenting in the life of individuals with disability.

As suggested in the analysis, parenting plays a normalizing and rehabilitating role in the life of individuals with disability. It is a source of profound positive experiences which form bonds between family members (especially between the spouses) and satisfaction from overcoming one’s limitations. It allows the individual to fulfill their needs (among others: love and self-realization) and develop the sense of self-esteem. It plays a vital role in developing a positive identity, a sense of femininity, motivation and the meaning of life [7,3 20,4 27]. The decision about becoming a mother is sometimes taken ‘to spite’ negative opinions of the environment, but its source lies not so much in the need to manifest one’s own opinion as in a deep psychological need. The child becomes the highest value, “more important than the mother’s own health” [7]. Women with muscular dystrophy in a study by O. Prilleltensky [6] said that pregnancy was a proof that their organism functions normally (at least some of its parts). Pregnant women with disability who took part in in-depth interviews in a study by R. Mayes et al. [22] talked about the positive experience of motherhood (including pregnancy) despite the negative attitudes of their close ones. For those women pregnancy was a source of an amazing feeling of their child developing in their body—an incomparable and unique experience. Each pregnancy was exciting and special. Separating themselves from their own experiences, they were determined to become better mothers than their own mothers were. Their responses encapsulate the essence of motherhood:

Becoming a mother is special: you get to hold this little baby in your arms, you get to hold it for the rest of your life. Each baby is special. […] when you feel the baby move inside you, you realize there is a real live human being in there, how could anyone kill that human being in your tummy [22, p. 127].

61 % of women with disability in Korea had positive experiences from the time of pregnancy [21].^5^

‘Disabled parenting’, just as any other parenting, is a source of various experiences, both positive and negative ones. They coexist, like they do in able-bodied parents’ life, and influence the very personal way in which one experiences parenting. Authors who conduct studies on women with disability write about a certain ambivalence of emotional experiences, “on the one hand there is this great joy of motherhood, on the other, there is uncertainty whether the child will suffer because of his/her mother’s disability” [7, p. 52]. On the basis of her research (using in-depth interviews) with 8 mothers who suffer from disability and chronic diseases, R. S. Farber [28] writes about a paradox referring to the work by E. Larson devoted to mothers raising children with disability. Women with disability who are mothers share similar experiences and the sense of motherhood with other mothers. It is reflected in the feeling of sadness resulting from their otherness, and at the same time they accept the uniqueness of motherhood (different because of their disability). The paradox may stem from the fact that these mothers cannot integrate their disability with the process of becoming a parent, especially if the disability stands in contrast to the ideal image of parenting [28]. This phenomenon quite aptly shows the nature of parenting, where difficulties are interwoven with successes.

Literature on the subject does not devote much attention to fathers with disability or their experiences. Studies by M. Kilkey and H. Clarke [20], and M. Kilkey [29]^6^ with fathers with disability yielded a multifaceted picture of these fathers’ experience. Men with disability who cannot perform professional roles are even more engaged in fulfilling their parental role, especially in its functional dimension. Such fathers are called stay-at-home dads and hands-on. They appreciate the time they can devote to their child. However, they also experience conflicts whose basis lies in the image of the father as the breadwinner held by the society:

I loved spending time with my son and staying at home but I felt it wasn’t the right thing. I just felt I should be the main breadwinner […] My life drastically changed; I wouldn’t have even considered being a stay at home dad prior to my accident but I think the fact that there was a possibility it just made me feel less of a I suppose it sounds really bad against women, but it made me feel less of a man being the person who was provided for instead of providing for […] One of the hardest things has been not as a disabled dad but as a stay at home dad; it’s hard being accepted in some circles [29, p. 25-26].

It is important to notice, though, that the authors of the cited words are men who became disabled in their adult life. Disability made it impossible for them (for various reasons) to stay professionally active, but it did not prevent them from fulfilling their family roles. Still, experiencing the described tensions affects fathers’ psycho-physical wellbeing and the quality of their parental role (perhaps in the long-term perspective). Therefore, discussing fathers’ experiences, one should bear in mind the many spheres of life where these men function; parenting is only one area of interest and actions, for some more and for others less significant [Rapaport, in 20]. A similar conclusion may be drawn regarding mothers who can take on many roles during their lifetime. Experience gained therefrom is interrelated.

The problems mentioned by parents of children with disability are often caused by factors that lie ‘outside’: they are caused by the way the economic and social environments are organized to suit the possibilities and needs of able-bodied persons. Parents with disability, and—in consequence—their families as well, experience poverty because they cannot find employment and because of high expenses (for the treatment), limited participation in public life, difficulties in accessing public places, limitations in access to information, and social isolation [2, 5, 21, 23, 26, 30]. Discussions on the impossibility to give proper care or spend leisure time in a similar way to how the able-bodied persons do [8], are in fact, discussions on the difficulties founded in the unfavorable organization of the environment and the lack of support (appropriate in its nature and scope) [7]. Undoubtedly, one cannot decidedly rule out the impact of personal factors. Still, their limiting influence should be discussed within the scope of the abovementioned environmental aspects. C. Malacrida [2]^7^ offers examples of this way of understanding problems. In her study, women with physical disability talked about physical barriers and their inability to cross them. These barriers limit the developing child’s access to a more open environment and make it more difficult to provide him/her with a wider scope of activities. The discussed barriers made one of the women sign up her able-bodied child to a special needs kindergarten, since it was the only one suited to this mother’s needs. C. Malacrida also writes that “if any family member will be disabled, it will be a child” [2, p. 479].

Parents with disability are not passive when faced with difficulties. Parenting seems to help them develop resources (mainly mental) they were lacking in before. L. Marszałek’s research results [8] on women with disability who realize their family roles (the third group) show that the majority of those women have positive self-esteem: 77 % in their conjugal role, 84 % in their parental role, and none of the respondents feel incompetent. The sense of competence and positive self-esteem in one’s parental role are related to higher perseverance and creativity, as suggested by C. Malacrida [2]. One of the mothers with physical disability who was raising her child alone and was having trouble providing the child with interactions in the outside world (not at home) offered parents from the neighborhood free caretaking of their children so that her own child could spend time with his/her peers. The same paper presents the findings to prove how determined mothers with intellectual disability are: having been deprived of the right of custody, they tried for many years (in one case, for 11 years) to raise their level of parental competence and be reassessed as competent in this respect.

Parents with disability are afraid of negative assessment [23].^8^ C. Thomas’s interview analysis show that this fear accompanies mothers with disability all the time [24]. If they are assessed negatively by specialists (doctors, midwives, social workers, and environmental nurses) they may be subjected to procedures depriving them of their right of custody. This fear strengthens the need to show that they are effective in realizing their parental role at any cost. Mothers do not look for support outside, since it would imply their incompetence. As a result of these efforts and the sense of loneliness in managing on their own, they experience a decrease in emotional and physical wellbeing [24]. In a study by H. McFarlane [23, p. 173] a mother with visual impairment admitted:

At the time I felt that my ability to look after and to care for my son was being questioned and scrutinized. I found it a real pressure, not knowing when she would just turn up… I made sure the baby and the house were immaculate all the time, but that was at great personal cost to myself. I mean I think within a very short space of time, realizing that I felt quite depressed and that I didn’t feel confident.

## Conclusion

Bearing in mind the issues discussed above, it may be concluded that the social context for disabled parenting is more often limiting than supporting or promoting development. The social discourse of disabled parenting on the professional and nonprofessional level concentrates on the difficulties and limitations which result from impairments. Therefore, it is set within the medical model of disability and, as such, it contradicts the tendencies promoted nowadays, such as normalization, integration, and postulates for a civic model of life with full rights, privileges and duties for everyone.

One might ask whether, in the context of the possible difficulties related to the mother’s and the child’s health, the risk of inheriting disorders, the decision about parenthood is not a sign of irresponsibility. The answers will vary. And still, parenthood is everyone’s right and a sign of free will. Moreover, specialists’ predictions about the genetic risk are not always well-founded (e.g. in the case of intellectual disability), not to speak of opinions expressed by laypeople. In this context, the question whether individuals with disability can (should) be parents seems groundless, and the only reasonable answer is, “Why not?” It seems that, in the light of the discussed tendencies (widely debated in various environments of education, rehabilitation and support), the right question to ask is, “How can we support individuals with disability in their preparations for parenting and later, when they start their family?”

The general remarks in support of parents with disabilities that come to mind after the above analysis are, as follows:

1. Although parenting marks a certain stage of human life and it constitutes a complex role that is voluntarily taken on, or imposed (by life situation), what is important is the nature of experiences gathered throughout one’s whole life, especially the ones related to socialization. They are the source of certain competences: personal (e.g. self-determination, positive self-image in various spheres, the ability to set life goals appropriate to one’s possibilities, and the motivation to reach them, managing on one’s own), social (social competence) and practical ones (learning skills important for becoming at least partially independent in various spheres of life), which can be viewed as the ‘equipment’ that everyone takes for their journey into adulthood with its roles and tasks. It should be stressed that being prepared is important irrespective of whether the individual will take up family roles (marital and parental) or not. Parenting and marriage should not be treated as unconditional indicators of normalizing life of individuals with disability, but as its aspects which are a matter of a voluntary and conscious choice.
2. The essence of disabled parenting lies in the interaction of individual and environmental factors. As suggested above, the impact of impairment (its scope and complexity) cannot be overlooked. Still, its influence on the quality of the fulfillment of parenting duties is a product of cooperation with early intervention and support [26].
3. Each instance of professional intervention must by subjective and individualized. As far as possible, it is the parents who should be the coordinators of support. It gives them a sense of responsibility for the child’s wellbeing [6].

Conclusions reached in point 1–3 are important for rehabilitation practice, especially for:

- parents and guardians of persons with disability, professionals (psychologists and pedagogues) who are an important source of socialization experience which leads to developing the life skills described in point 1. It is important for parents, guardians and professionals to see the significance of taking up various social roles, including parenting and marital roles for the quality and normalization of disabled individuals’ lives. Coherence of socialization methods used by the abovementioned persons who are present in the lives of individuals with disability is also important.
- professionals who offer psychological, functional and financial support in various institutions to persons with disability who take up family roles. Cooperation between various resorts (e.g. state aid and health) which guarantees extensive support. Positive attitudes of professionals working in institutions, accepting the needs, difficulties, limitations, but also being able to see personal resources of individuals with disability.
- organizations for persons with disability. They focus predominantly on parents of individuals with disability. While they should be gradually broadening their offer to include support for individuals with disability who enter adulthood and realize various social roles in their adult life (i.e. family and professional roles).

All the presented analysis are based on studies carried out in various socio-cultural and economic contexts. Conclusions presented here should, therefore, be taken cautiously, even more so, since they derive from qualitative data from subjective experiences gained in specific contexts of living environment and socialization environment of parents with disability. Still, qualitative data is the most useful in analyzing the subject raised in this paper. Richness of experience related to this sphere of life, and its entanglement in contextual factors may be verified only by biographical narrative methods.

Further research into the field should include concepts such as parenting experience of persons with disability over the course of their life (longitudinal study); availability of social and personal resources and realizing family roles; functioning in family roles and professional roles (e.g. role conflict and role enrichment); adapting to disability and the quality of realizing family roles.

## Footnotes

1 A mother who took part in a study by H. McFarlane [23] said that when a child was sitting on her laps, people were asking, “Oh, and whose baby is this?”, and when she replied that it was hers, “Oh! What are you doing with a baby” and “oh, I didn’t think you would be able to manage having a baby”.

2 Semi-structured interviews were used in studies with 17 women with disability who have a small child or who are pregnant.

3 On the basis of analysis of 156 diaries of women with disability who took part in the contest.

4 On the basis of in-depth interviews with 60 fathers with disability.

5 On the basis of structured interviews carried out with 410 mothers aged 18–50.

6 Studies using semi-structured interviews with 30 fathers with physical/sensory disability or learning difficulties.

7 Semi-structured interviews were conducted with 43 women with physical, sensory, mental or cognitive impairments, who have children aged 18 or under.

8 Narratives of 27 women with disability.
